# Supplementary material for: Effects of Nox4 upregulation on PECAM-1 expression in a mouse model of diabetic retinopathy
Source: PLoS One. 2024 May 15;19(5):e0303010. doi: 10.1371/journal.pone.0303010 (PMC11095704; doi:10.1371/journal.pone.0303010)
Supplement: S1 File — (PDF) [file pone.0303010.s001.pdf]

Fig. 1B

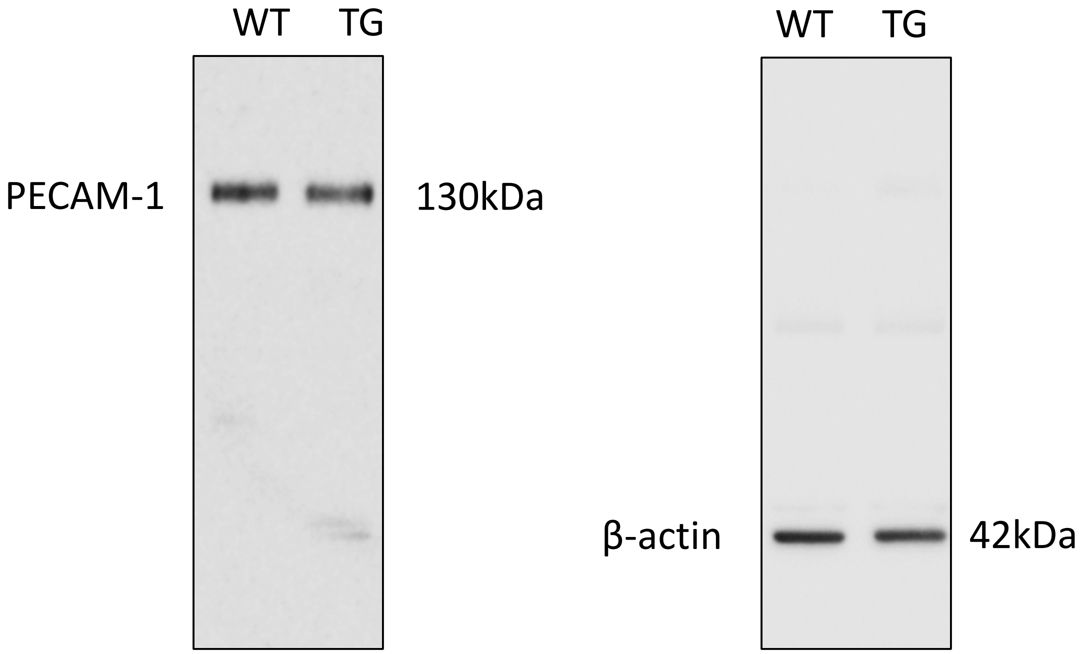

Fig.3B

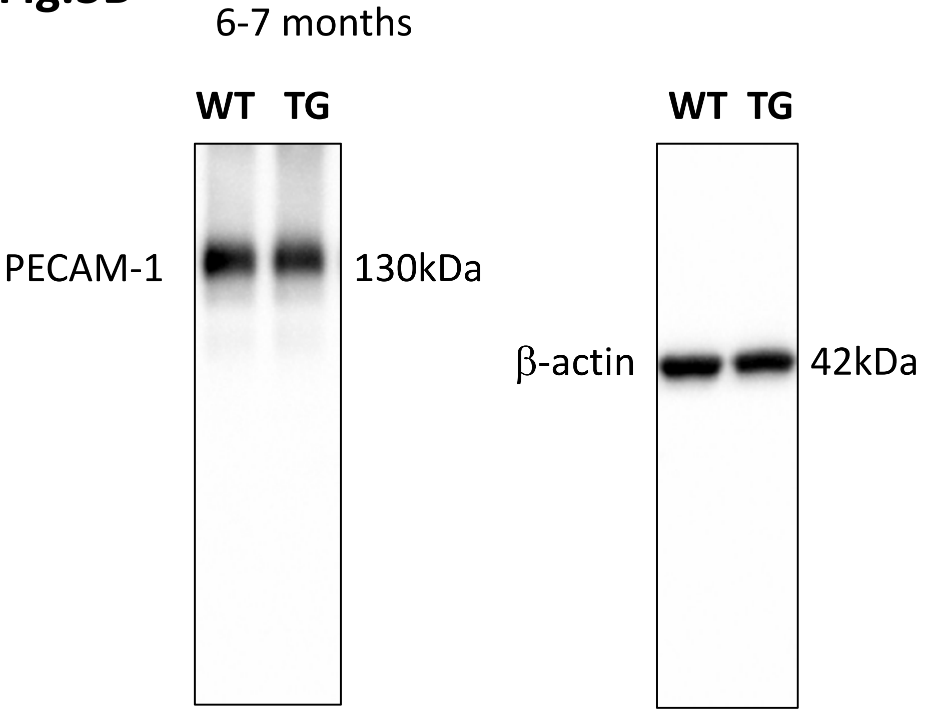
6-7 months
WT TG
WT TG
PECAM-1
130kDa
β-actin
42kDa

Fig. 3D

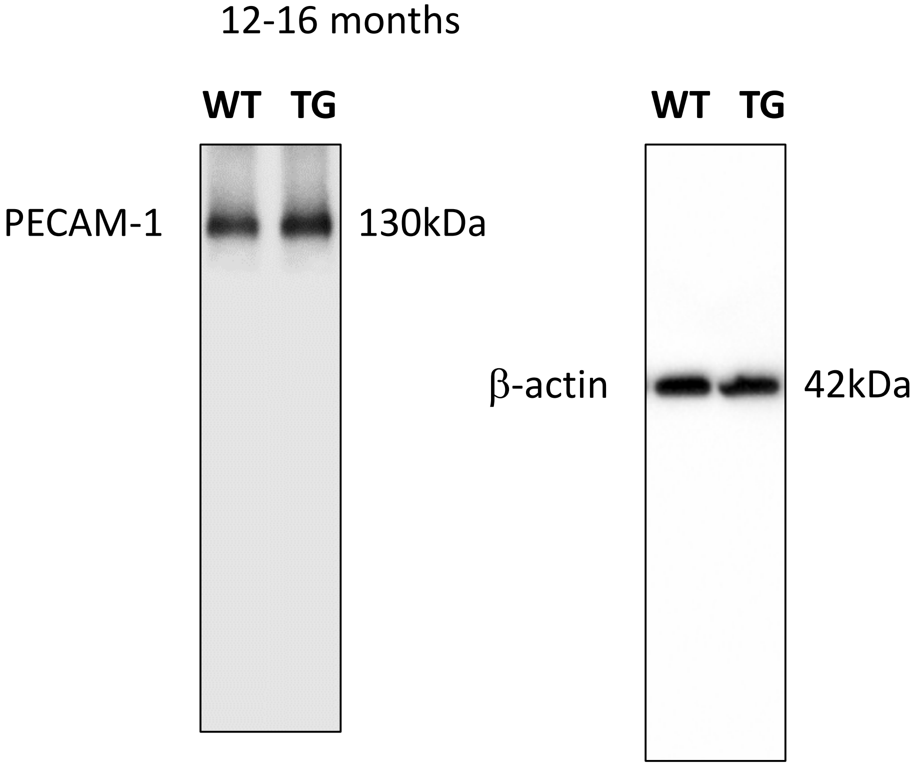
12-16 months
WT TG
WT TG
PECAM-1
130kDa
β-actin
42kDa

Fig. 4B

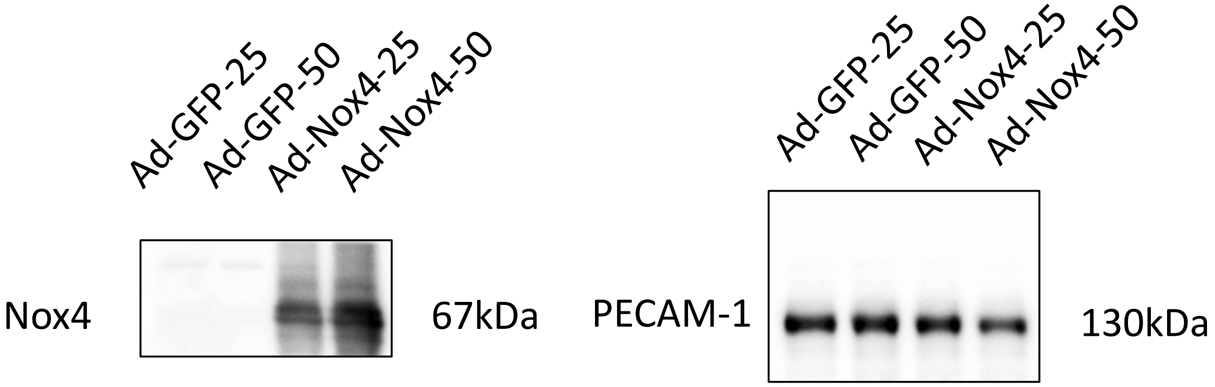

(membrane was cut before incubating in primary antibody)

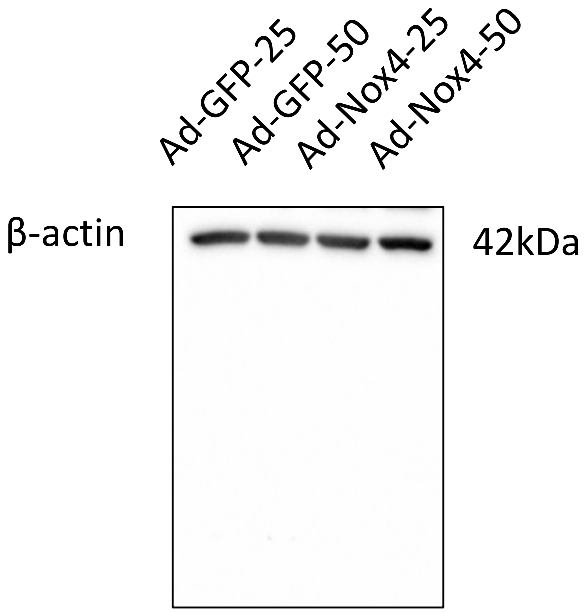

(membrane was cut before incubating in primary antibody)

Fig. 5B

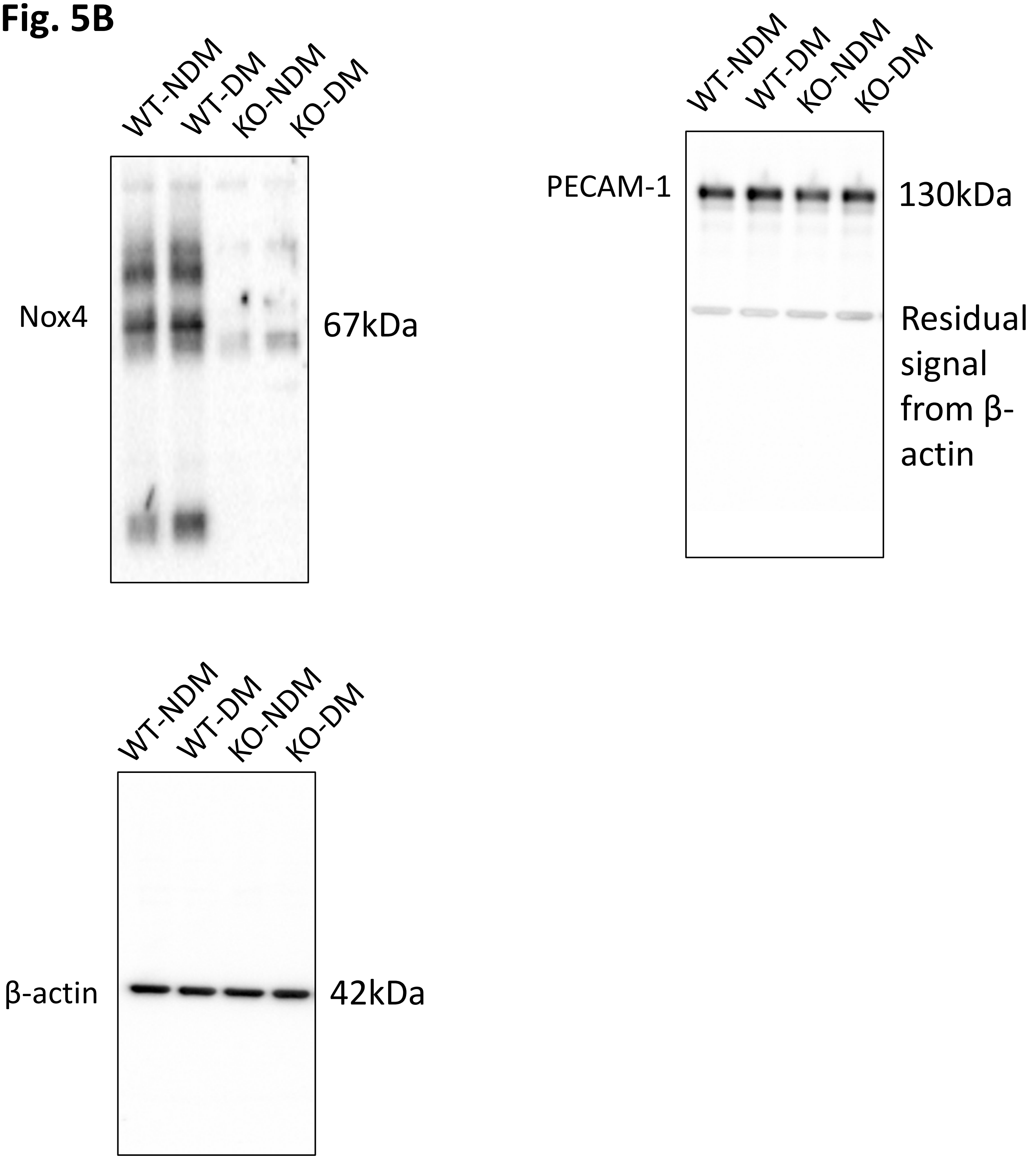

WT-NDM
WT-DM
KO-NDM
KO-DM
Nox4
67kDa
PECAM-1
130kDa
Residual signal from β-actin
β-actin
42kDa
