# Supplementary material for: Effects of Nox4 upregulation on PECAM-1 expression in a mouse model of diabetic retinopathy
Source: PLoS One. 2024 May 15;19(5):e0303010. doi: 10.1371/journal.pone.0303010 (PMC11095704; doi:10.1371/journal.pone.0303010)
Supplement: Attachment — Submitted filename: plosone rebuttal-3-27-24.pdf [file pone.0303010.s005.pdf]

Reviewer 1

Not all the self-proteins are safe, immunology is a complicated system. A great deal of autoimmunity progressions and associated diseases have been reported. The human Nox4 protein might bind with some mice proteins to form epitopes. I have attended a number of clinical trials of protein drugs, and in animal work, the proteins/antibodies targeting human proteins usually don't work well in mice/rats/rabbits/monkeys.

Although transgenic animals have been used in the research of human diseases; the animal molecules work in the progression of a certain disease in animal models. My suggestion is to try the knock down/knock out/overexpression of mice Nox4 in mice model.

If the authors do not want to design the experiments of mice Nox4, the title should be clarified as "Effects of Human Nox4 Upregulation on PECAM-1 Expression in a Mouse Model of Diabetic Retinopathy". Obviously, human Nox4 could not displace mice Nox4 in associated signal axes; and then this work seems to make no sense.

Response to Reviewer: We very much appreciate the reviewer's further comments. We agree that although humanized animal models have been widely used and are becoming a powerful model in biomedical research, they do have some limitations. We also appreciate the reviewer's constructive suggestions of "try the knock down/knock out/overexpression of mice Nox4 in mice model". The knockout mice lacking endogenous mouse Nox4 in endothelial cells, referred to as "EC-specific Nox4 conditional knockout (cKO) mice", were used in this study. Results from EC-Nox4 cKO mice were presented in Figure 5.

Reviewer 2

After carefully reading all the comments and responses, I think I agree with another reviewer's comments. Human body and mouse are two different machines; human and mice Nox4 are different components in these two machines, although these two molecules shared high similarity, they are not the same. The conclusion of this manuscript is that overexpression of Nox4 increases PECAM-1 mRNA but has no effect on its protein expression in the mouse retina, BMECs, or HRECs. Could the authors try to knock down or overexpress mice Nox4 in eye in mice model using AAV vector? In addition, as reported, most of the signal pathways are crosslinked, and PECAM-1 should not be the only target of Nox4, the authors should screen the direct targets of Nox4 via co-IP or other associated protocols.

Response to Reviewer: Thank you for taking time to carefully read all the comments and responses. We also appreciate the reviewer's valuable suggestions--"try to knock down or overexpress mice Nox4 in eye in mice model using AAV vector and screen the direct targets of Nox4 via co-IP". However, screening the direct targets of Nox4 is beyond the scope of the current manuscript.
