# Supplementary material for: Effects of Nox4 upregulation on PECAM-1 expression in a mouse model of diabetic retinopathy
Source: PLoS One. 2024 May 15;19(5):e0303010. doi: 10.1371/journal.pone.0303010 (PMC11095704; doi:10.1371/journal.pone.0303010)
Supplement: Attachment — Submitted filename: General Comment.docx [file pone.0303010.s002.docx]

Manuscript Number PONE-D-23-36248

Minimal Effects of Nox4 Upregulation on PECAM-1 Expression in Diabetic Retinopathy

General Comment：

Wang et al investigate the impact of Nox4 upregulation on PECAM-1 expression in mouse retinas and brain microvascular endothelial cells (BMECs) using endothelial cell-specific human Nox4 transgenic (TG) mice and endothelial cell-specific Nox4 conditional knockout (cKO) mice. The manuscript is well written, well presented, and presents convincing evidence of not support a significant role of Nox4 in the regulation of PECAM-1 expression in the diabetic retina and endothelial cells. However, there are some major problems need to be addressed.

Major points:

1. The breakdown of endothelial junction proteins, such as ZO-1, occludin, and claudin, has been shown to contribute to vascular hyperpermeability in DR. This study, however, aims to investigate the role of Nox4 in relation to PECAM-1. It would be helpful to know if there is any preliminary data, such as RNA-sequencing data of endothelial cells with NOX4 interference, that could support this investigation.
2. Platelet endothelial cell adhesion molecule 1 (PECAM-1) is a cell surface molecule that is highly expressed in endothelial cells, but also expressed in various circulating cells, such as platelets, monocytes, neutrophils, and certain T cells. In this study, was vascular perfusion performed in animal experiment to eliminate the interference of the blood cells ?
3. In figure 1 and figure 2, please point out the age of the TG mice.
4. In figure 1 and figure 2, this study was lack of the result of retinal vascular permeability of TG mice. There was no difference in PECAM-1 expression between WT and TG mouse retinas in this study. Is this possibly related to the absence of retinal vascular leakage in this particular group of TG mice?
5. In figure 2D, is PECAM-1 specific to EC? In retinal cryosections derived from WT and TG mice, the PECAM-1(green) also appears to be expressed in other tissues.
6. In figure 1 and figure 2, quantitative intensity analysis of PECAM-1 / IB4 ratio in the immunofluorescence staining of retinal wholemounts is suggested.
7. Please show the quantitative intensity analysis of PECAM-1 in figure 3A, It appears that the intercellular junctions in the TG group are thicker compared to the WT group.
8. PECAM-1 has been shown to decrease in the diabetic retina. However, the slight increases in both mRNA (Fig. 5A) and protein (Fig. 5B&5C) levels of PECAM-1 in diabetic WT BMECs were observed compared to non diabetic WT controls in the present study. Please explain the inconsistency.
