# Supplementary material for: Effects of Nox4 upregulation on PECAM-1 expression in a mouse model of diabetic retinopathy
Source: PLoS One. 2024 May 15;19(5):e0303010. doi: 10.1371/journal.pone.0303010 (PMC11095704; doi:10.1371/journal.pone.0303010)
Supplement: Attachment — Submitted filename: Response to Reviewers-PlosONE-12.15.docx [file pone.0303010.s003.docx]

December 15, 2023

RE: PONE-D-23-36248

Dear Reviewers,

Thank you so much for carefully evaluating our manuscript and providing constructive comments to improve our manuscript. Following your suggestions, we have revised the manuscript and provided additional information/analyses as requested. We feel that these changes have greatly strengthened the manuscript. Below we provide point-to-point responses to each of your comments and summarize the changes we made to the manuscript.

**Reviewer #1**

1. *In the authors' previous work, diabetes brought the upregulation of Nox4, which could lead to the increasing of retinal vascular leakage. And in this work, PECAM-1 was selected as a candidate, however, how was it screened? Was there any supporting information? The direct interaction between Nox4 and PECAM-1? Are there any intermediate steps？*

**Response:** Thank you for your comments. As discussed in the manuscript (Introduction and Discussion sections), PECAM-1 is a cell adhesion molecule highly expressed in cell-cell junctions of endothelial cells. It regulates cell junction integrity and thus contributes to endothelial barrier function. Previous studies demonstrated that PECAM-1 is downregulated in the diabetic retina and downregulation of PECAM-1 contributes to increased vascular permeability in diabetic retinopathy. Our prior work has shown that Nox4 upregulation in endothelial cells leads to increased vascular leakage. Therefore, in this study, we sought to determine whether Nox4’s effect on vascular leakage was through PECAM-1 downregulation. To our knowledge, there is no published study showing a direct interaction between PECAM-1 and Nox4. Our results from the current study do not support a regulatory role of Nox4 in PECAM-1 expression in retinal endothelial cells during diabetes. Our ongoing studies are investigating the effects of Nox4 on regulations of other junctional complexes such as tight junctions in retinal endothelial cells and DR.

*2. As PECAM-1 could not be regulated by Nox4, what's the exact downstream target of PECAM-1?*

**Response:** The downstream target and upstream regulators of PECAM-1 have been investigated in several recent studies (see Discussion section). In diabetic retinopathy, increased peroxynitrite activates MMP2, which interacts with PECAM-1 protein resulting in its degradation (Eshaq RS and Harris NR, IOVS, 2019;60:748-60; cited as Ref. 4). Hyperglycemia and inflammatory factors (TNF-α and IFN-γ) can also reduce PECAM-1 protein level in retinal endothelial cells by increasing its ubiquitination and proteasomal degradation (Eshaq RS and Harris NR, Microcirculation. 2020;27:e12596; Eshaq RS and Harris NR, Microcirculation. 2021;28:e12717; cited as Refs. 20 and 21). As to the downstream target, the authors showed that PECAM-1 can interact with β-catenin and loss of PECAM-1 leads to increased vascular permeability through reducing β-catenin levels in retinal endothelial cells (Eshaq RS and Harris NR, Microcirculation. 2021;28:e12717; Ref. 20). In addition, PECAM-1 itself is an adhesion junctional protein regulating endothelial cell junctional integrity.

*3. The human and mice Nox4s shared relatively high sequence identity, however, there might be some epitopes. Why the human Nox4 transgenic mice was used? If the authors hope to investigate the effects of Nox4 overexpression in mice, the mice Nox4 overexpression plasmid or AAV should be used.*

**Response:** Thank you for your comments. The humanized Nox4 transgenic mouse line was generated in our previous study (Tang, Xixiang, et al. "Sustained Upregulation of Endothelial Nox4 Mediates Retinal Vascular Pathology in Type 1 Diabetes." Diabetes, 2023, 72: 112-125; cited as Ref. 11). Human Nox4 overexpression was used because of its relevance to human disease of DR. In this study, we have shown that Nox4 overexpression induces endothelial cell dysfunction, focal vascular leakage, and capillary degeneration, which recapitulate the major vascular pathology of DR. Given our goal is to investigate the role of Nox4 in endothelial cells in DR, overexpression of Nox4 by plasmid or AAV via intravitreal injection is not feasible, because of the poor transfection/transduction efficiency of retinal endothelial cells.

**Reviewer #2**

*1. The result of this work is Nox4 could not regulate PECAM-1 in DR. So, why Nox4 and PECAM-1 were investigated? What could be affected by Nox4 in the progression of DR?*

**Response:** Please see responses to Reviewer#1, Comment 1, and response to Reviewer #1, Comment 3.

*2. Are there any intermediate factors between these two molecules?*

**Response:** Please see responses to Reviewer#1, Comment 1.

*3. Why a human Nox4 transgenic mice model was used? Although Nox4 is conserved in human and mice, their sequences are not same. If the authors hope to investigate the effects of Nox4 overexpression and knockdown in mice, the mice Nox4 should be overexpressed but not human Nox4.*

**Response:** Please see responses to Reviewer#1, Comment 3.

**Reviewer #3**

Major points:

*1.The breakdown of endothelial junction proteins, such as ZO-1, occludin, and claudin, has been shown to contribute to vascular hyperpermeability in DR. This study, however, aims to investigate the role of Nox4 in relation to PECAM-1. It would be helpful to know if there is any preliminary data, such as RNA-sequencing data of endothelial cells with NOX4 interference, that could support this investigation.*

**Response:** Thank you for the comment. We did not perform an RNA-sequencing study of endothelial cells with Nox4 interference. As you mentioned, the breakdown of endothelial tight junction proteins contributes to vascular permeability in DR. Loss of PECAM-1 has also been shown to increase vascular permeability (Ref. 20). We feel that the regulation of PECAM-1 is less intensively studied compared to tight junction proteins such as *ZO-1, occludin, and claudin*. Thus, in the present study, we determined whether Nox4 upregulation alters PECAM-1 expression in endothelial cells. Our ongoing studies are investigating the effects of Nox4 on the regulation of other junctional complexes focusing on tight junction proteins in retinal endothelial cells and DR.

*2.Platelet endothelial cell adhesion molecule 1 (PECAM-1) is a cell surface molecule that is highly expressed in endothelial cells, but also expressed in various circulating cells, such as platelets, monocytes, neutrophils, and certain T cells. In this study, was vascular perfusion performed in animal experiment to eliminate the interference of the blood cells?*

**Response:** Thank you for the valid point. We did not perform vascular perfusion in the animal experiment. We used isolectin B4 to label endothelial cells in retinal vessels and observed PECAM-1 well colocalized with isolectin B4, suggesting its localization to endothelial cells. To further explore the regulation of PECAM-1 in endothelial cells by Nox4, we isolated and cultured the brain microvascular endothelial cells (BMECs) from TG mice. Thus, we believe that these measures have helped in excluding the confounding factors from the circulating cells.

*3.In figure 1 and figure 2, please point out the age of the TG mice.*

**Response:** We have included the age information for Fig. 1 and Fig. 2 in the figure legends.

4.In figure 1 and figure 2, this study was lack of the result of retinal vascular permeability of TG mice. There was no difference in PECAM-1 expression between WT and TG mouse retinas in this study. Is this possibly related to the absence of retinal vascular leakage in this particular group of TG mice?

**Response:** Please see the response to Reviewer #1, Comment 3. In a previous study (Ref. 11), we demonstrated increased vascular leakage and capillary degeneration in the TG mice.

*5. In figure 2D, is PECAM-1 specific to EC? In retinal cryosections derived from WT and TG mice, the PECAM-1(green) also appears to be expressed in other tissues.*

**Response:** Thank you for the comment. As mentioned in the Introduction, PECAM-1 is highly expressed at endothelial cell junctions but is also present on the surface of other circulating cells including granulocytes, monocytes, and platelets. In our experiments using immunohistochemistry of retinal cryosections, we observed nonspecific background fluorescence of anti-PECAM-1 antibody outside of IB4-stained vasculature. However, it is noteworthy that PECAM-1 immunostaining in the retinal whole mounts did not exhibit significant background fluorescence. This observation supports that the increased background signal is likely unrelated to extravascular PECAM-1 but rather caused by an unknown factor intrinsic to the process of preparing the cryosection samples.

*6. In figure 1 and figure 2, quantitative intensity analysis of PECAM-1 / IB4 ratio in the immunofluorescence staining of retinal wholemounts is suggested.*

**Response:** Thank you for the valuable suggestion. In our analysis of PECAM-1 immunofluorescence staining in retinal whole mounts, we employed IB4 staining as a qualitative control to demonstrate the colocalization of PECAM-1 within the retinal vasculature. Thus, we did not undertake a quantitative analysis of IB4 fluorescence. IB4 was used as a marker of endothelial cells. Therefore, we respectfully believe it would not be beneficial to use its fluorescence as a quantitative control value in the form of PECAM-1 / IB4 ratio.

*7.Please show the quantitative intensity analysis of PECAM-1 in figure 3A, It appears that the intercellular junctions in the TG group are thicker compared to the WT group.*

**Response:** Thank you for the suggestion. We have added the quantitative intensity analysis of PECAM-1 for Fig. 3A. The result showed no statistical differences in PECAM-1 intensity between WT and TG groups (n= 4-5 mice/group, t-test, P>0.05).

*8. PECAM-1 has been shown to decrease in the diabetic retina. However, the slight increases in both mRNA (Fig. 5A) and protein (Fig. 5B&5C) levels of PECAM-1 in diabetic WT BMECs were observed compared to non diabetic WT controls in the present study. Please explain the inconsistency.*

**Response:** We have discussed extensively the inconsistency in PECAM-1 changes in the diabetic retina, reported in the present and previous studies. Please see the Discussion section, lines 324 – 363.
