# Supplementary material for: Effects of Nox4 upregulation on PECAM-1 expression in a mouse model of diabetic retinopathy
Source: PLoS One. 2024 May 15;19(5):e0303010. doi: 10.1371/journal.pone.0303010 (PMC11095704; doi:10.1371/journal.pone.0303010)
Supplement: Attachment — Submitted filename: plosone rebuttal-final 2-7-24.docx [file pone.0303010.s004.docx]

**Reviewer 1, Question #1:** *The title revealed a negative result that Nox4 upregulation could not significantly affect the expression of PECAM-1 in DR. Could the title be reconstructed in a more appropriate sentence?*

Response to Reviewer: Thank you very much for this insightful suggestion. We re-formulate the title and change it to “Effects of Nox4 Upregulation on PECAM-1 Expression in a Mouse Model of Diabetic Retinopathy”. We believe that it is more suitable and concise now.

**Reviewer 1, Question #2:** *Using human Nox 4 mice is a serious problem, although the sequence identity between human and mouse Nox4 was ~90.8%, human Nox4 is still a foreigner and potential antigen in mouse body, which might bring with undesirable immunological reactions. In the authors' response, DR is a human disease and human Nox4 was used. However, the DR process was built in a mice model, it's also a mice disease.*

Response to Reviewer**:** We totally understand and appreciate your concern regarding the use of human Nox4 transgenic mice. To address your question, we have consulted experts in genetic animal models and have searched the literature extensively in PubMed and Google Scholar. Transgenic mice overexpressing human genes or cells have been valuable pre-clinical models to study human diseases for over 30 years. No report demonstrates undesirable immunological reactions to human gene in transgenic mice. This is because once a human gene is inserted into the mouse genomic locus, it is considered as an integral part of the mouse genome. The protein expressed in the mouse tissue will be considered as its own protein, which will not cause an immune response. Please refer to Ref. 1 and Ref. 2 for detailed information on how B cell- and T cell-mediated self-tolerance is established. As to the human Nox transgenic mice, human Nox4 is expressed in Tie2-expressing cells at the embryonic stage. In addition, Nox4 is an intracellular protein that is not exposed to the innate immune system. Therefore, we do not expect an immune response caused by human Nox4 in the transgenic mice.

**Reviewer 2, Question #1:** *DR is a human disease, however, in this work, the whole DR progression was in mice model, using a human element in a whole mice progression is not appropriated.*

Response to Reviewer: We understand your concern and thank you for comments. Animal models are widely employed in scientific research and are essential tools for the study of most, if not all, human diseases. Due to logistical and ethical restrictions to study human genes in human subjects, genetically humanized mice have become increasingly important as animal models for the study of human diseases (Ref. 3). Like most proteins, Nox4 is not 100% conserved between human and mouse. Specifically, there is a ~90.8% sequence homology between human and mouse Nox4. The differences in these orthologous sequences may have functional consequences (Ref. 4). In addition, human-specific genes and the encoded proteins are likely to function as regulators of cell signaling events specific to human diseases (Ref. 5). Therefore, the use of humanized NOX4 mice is an appropriate and valuable model in studying human disease like DR.

**Reviewer 2, Question #2:** *Although human and mice Nox4 show homologous sequence identity, there are still some fragments which could be recognized as epitopes and may lead to immunological reactions. In the clinical diagnosis and fundamental research of DR, local inflammation is also one of the important features.*

Response to Reviewer: We totally understand and appreciate your concern. Please refer to Response to Reviewer 1, Question #2.

**References**

1. Nemazee D. Mechanisms of central tolerance for B cells. Nat Rev Immunol. 2017;17(5):281-294. doi: 10.1038/nri.2017.19. PMID: 28368006.

2. Xing Y, Hogquist KA. T-cell tolerance: central and peripheral. Cold Spring Harb Perspect Biol. 2012;4(6):a006957. doi: 10.1101/cshperspect.a006957. PMID: 22661634.

3. Walsh NC, Kenney LL, Jangalwe S, Aryee KE, Greiner DL, Brehm MA, Shultz LD. Humanized Mouse Models of Clinical Disease. Annu Rev Pathol. 2017; 12:187-215. doi: 10.1146/annurev-pathol-052016-100332. PMID: 27959627.

4. Devoy A, Bunton-Stasyshyn RK, Tybulewicz VL, Smith AJ, Fisher EM. Genomically humanized mice: technologies and promises. *Nat Rev Genet*. 2011;13:14-20. doi:10.1038/nrg3116

5. Stahl PD, Wainszelbaum MJ. Human-specific genes may offer a unique window into human cell signaling. Sci Signal. 2009;2(89):pe59. doi: 10.1126/scisignal.289pe59. PMID: 19797272.
